# A Framework for Interactive Medical Image Segmentation Using Optimized Swarm Intelligence with Convolutional Neural Networks

**DOI:** 10.1155/2022/7935346

**Published:** 2022-08-24

**Authors:** Chetna Kaushal, Md Khairul Islam, Sara A. Althubiti, Fayadh Alenezi, Romany F. Mansour

**Affiliations:** ^1^Chitkara University Institute of Engineering and Technology, Chitkara University, Punjab, India; ^2^Department of Information Communication Technology, Islamic University, Kushtia, Bangladesh; ^3^Department of Computer Science, College of Computer and Information Sciences, Majmaah University, Al-Majmaah 11952, Saudi Arabia; ^4^Department of Electrical Engineering, College of Engineering, Jouf University, Saudi Arabia; ^5^Department of Mathematics, Faculty of Science, New Valley University, El-Kharga 72511, Egypt

## Abstract

Recent improvements in current technology have had a significant impact on a wide range of image processing applications, including medical imaging. Classification, detection, and segmentation are all important aspects of medical imaging technology. An enormous need exists for the segmentation of diagnostic images, which can be applied to a wide variety of medical research applications. It is important to develop an effective segmentation technique based on deep learning algorithms for optimal identification of regions of interest and rapid segmentation. To cover this gap, a pipeline for image segmentation using traditional Convolutional Neural Network (CNN) as well as introduced Swarm Intelligence (SI) for optimal identification of the desired area has been proposed. Fuzzy C-means (FCM), K-means, and improvisation of FCM with Particle Swarm Optimization (PSO), improvisation of K-means with PSO, improvisation of FCM with CNN, and improvisation of *K*-means with CNN are the six modules examined and evaluated. Experiments are carried out on various types of images such as Magnetic Resonance Imaging (MRI) for brain data analysis, dermoscopic for skin, microscopic for blood leukemia, and computed tomography (CT) scan images for lungs. After combining all of the datasets, we have constructed five subsets of data, each of which had a different number of images: 50, 100, 500, 1000, and 2000. Each of the models was executed and trained on the selected subset of the datasets. From the experimental analysis, it is observed that the performance of K-means with CNN is better than others and achieved 96.45% segmentation accuracy with an average time of 9.09 seconds.

## 1. Introduction

**Figure 1:**
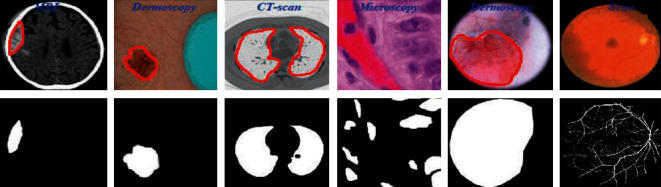
Medical image segmentation.

To advance the efficiency and accuracy of the medical diagnostic system, especially those that are distributed in complex areas (e.g., brain, skin, lung, and blood cancer classification), several live line diagnostic models (Bengio et al. [1]; Tiwari et al. [2]; Bhatt et al. [3]) work with image processing. The effectiveness of the detection and accuracy of the multidisciplinary medical data diagnostic system depends largely on the quality of the included images captured by a few techniques (Cetin et al. [4]) such as Magnetic Resonance Imaging (MRI) for brain data analysis, dermoscopic for skin, microscopic for blood leukemia, computed tomography (CT) scan images for lungs, etc. However, due to the uncontrollable lighting conditions and lots of noise availability during capturing, the illumination distributed on the surface of the medical images remains uneven, especially when backlight or fixed lighting conditions affect the diagnostic model (Bonabeau et al. [5]; Banks et al. [6]; Siva Raja and Rani [7]). It also leads to a comparison of the global low and local image and weak data in the black region and each aspect of the image plays a significant function in the examination of medical data. Therefore, preliminary processing is an important part of medical image classification because it plays an important role in computer-assisted medical diagnostic programs in different systems. Due to the position variability of the targeted regions, traditional hybrid segmentation technique such as Fuzzy Competitive Learning based Counter Propagation Network (FCPN) still works better than soft computing techniques [8]. Moreover, image classification is considered the most important process for medical imaging as it extracts the region of interest (ROI) from various data by semiautomatic or automatic process (Kennedy and Eberhart [9]). It classifies the image in areas based on a specific definition, such as the segmentation of damaged body parts or tissues in the local medical diagnostic system and the acquisition of boundaries and classification. There are some samples of medical image segmentations shown in Figure 1.

**Figure 2:**
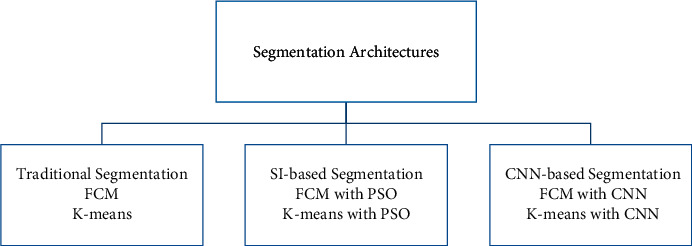
Proposed segmentation architectures.

For medical experts and researchers alike, performing good medical image segmentation is a difficult undertaking (Karaboga [10]). Many researchers, on the other hand, have previously attempted to create an effective algorithm for medical image segmentation in order to aid in the identification of various disorders and diseases. Therefore, in this research work, we present a comparative medical image segmentation framework using swarm intelligence with Convolutional Neural Networks (CNN). Mainly, three different medical image segmentation mechanisms are considered and studied, namely, Traditional Segmentation (Fuzzy C-means (FCM) and K-means), Swarm Intelligence (Particle Swam Optimization (PSO)), and CNN-based segmentation with traditional approaches, as shown in Figure 2 with subclasses of architectures.

Used traditional segmentation techniques are the type of unsupervised machine learning and useful to find out groups or different patterns in medical data. In general terms, it's an unsupervised activity that divides unstructured data into several groups based on their similarity and dissimilarity (Yang, 2009 [11]). The motivation behind the proposed scenario is given in further sections.

### 1.1. Motivation

Unsupervised clustering-based medical image segmentation is a default method that aims to collect a set of objects or pixels into subsets or collections by the background and front of the image. The goal is to create clusters or parts that fit inside but are very different from each other. In simple terms, pixels in the same category should be as similar as possible, and objects in the same category should be very different from those in another cluster. Some challenging factors given that gave us the motivation are as follows:

- The number of research articles available was large but lack of appropriate comparisons of Traditional medical image Segmentation, SI, and CNN-based segmentation.
- Medical data or image segmentation is a challenging task and still lots of improvements are needed to develop a better diagnosis system.
- Existing CNN-based models need a lengthy system-training period.
- Suffering from the over-fitting problems and need to solve such kind of problem regarding the medical diagnosis system that helps to detect the diseases in early stage. The overfitting problem in deep learning usually occurs when the image count is small in the target.
- The existing system had to be developed and updated in real-time scenarios.
- There are no studies that have established a single standard segmentation model for distinct picture types from various organs.

### 1.2. Contributions

Nowadays, medical image segmentation using clustering is a basic requirement for lots of purposes like abnormal region detection, automatic extraction, data organization, etc. In the segmentation of medical data, high-quality clustering techniques are critical. Thus, in this research, we proposed a comparative framework for medical image segmentation with SI as well as CNN technique and the main contributions are as follows:

- To study the existing medical image segmentation approaches with different algorithms.
- Develop a novel pre-processing for medical images like image quality enactment, hair removal from dermoscopic images, and blast nucleus improvement for microscopic images.
- To segment medical images, FCM and *K*-means are used as unsupervised machine learning approaches with PSO as swarm intelligence and CNN as a deep learning mechanism. A novel fitness function is presented here that replaces pixels to increase segmentation quality.
- To validate the proposed framework, performance parameters such as Precision, Recall, F-measure, Accuracy, Error, Matthews's Correlation Coefficient (MCC), Dice coefficient (DC), Jaccard Coefficient (JC), and time being calculated and related with existing works.

This research article deals with a comparative study for medical image segmentation and the rest of the article is systematized into different sections.  Section 2 illustrates the survey of related work, and the methodology of the proposed mechanism is described in Section 3. In Section 4, results and discussion based on the performance parameters are illustrated, and Section 5 concludes with recommendations for the future.

## 2. Literature Survey

Segmentation is widely used in various sectors such as split geographical regions, fruit from trees, flood for damage reports, recognition of traffic signs, and road collapses. Chouhan et al. [12] surveyed Computational Intelligence (CI) techniques to demonstrate the application of segmentation in the interdisciplinary research area. They discussed well-known CI techniques such as neural network, fuzzy logic, and genetic algorithms as well as they have also proved that CI-based approaches are cost-effective, time-saver, higher-efficient, and applicable in various engineering sectors [12, 13]. In 2021, researchers introduced an Internet of Things (IoT) device for automatic plant disease (galls) detection using the Fuzzy Based Function Network (FBFN) segmentation technique [14]. Similarly, a web-based tool was developed to identify mango leaf diseases such as Anthracnose using the RBF segmentation method. Thus, the segmentation techniques are universally implemented for different image-based detection techniques [15].

However, image segmentation is extensively used in human disease detection and diagnosis. For precise detection of the disease, initially, it requires identifying the region of interest from the captured images. In this study, a comprehensive description of the most important state-of-the-art medical image segmentation techniques is given. Here, we consider a mixed survey of segmentation for different types of medical data.

IMV-FCM, an enhanced multiscreen FCM clustering method, introduces a weighted adaptive learning technology to increase the flexibility of coordinating from diverse viewpoints. The algorithms might be able to learn from each view in an adaptive way that helps them better group brain tissue and deal with a noise like partial dimension distortions and grayscale that does not match up [16]. In 2018, Karegowda et al. [17] conducted research on the segmentation of brain tumor regions from MRI data. The authors conducted a comparative examination of FCM, Adaptive Regularized Kernel-based FCM approaches, PSO, and *K*-means and concluded that using PSO as swarm intelligence is a useful step. The results of the experiments showed that PSO-based segmentation is more accurate than FCM, Adaptive Regularized Kernel FCM, and *K*-means [17]. Arun Kumar et al. [18] created an improved automated approach for segmenting brain tumor regions and identifying them using K-means for the same objective. The goal of the authors was to improve the imaging enhancement at the pre-processing stage for precise brain tumor prediction [18]. Chander et al. [19] developed a framework for the segmentation of MRI images using K-means with Support Vector Machine (SVM) as the machine learning approach, and the overall accuracy was increased over earlier work [19]. In [20], the authors have discussed various methods such as traditional segmentation (Threshold, Fuzzy Theory, Region and edge detection), machine learning approach (KNN, Random Forest, SVM, Dictionary learning), and deep learning methods (CNN, FCM, Encoder/decoder). Although their analysis depicts that deep learning-based techniques such as FCM are superior to other traditional methods, they also include a supervised method that demands for manual labeling which required domain-specific knowledge [20].

In one of our previous studies, we have developed an IoT-based data collection system for skin lesions where we classified various skin lesions using deep learning-based ensemble algorithms [21]. For skin lesion segmentation, Yuan et al. [22] used the notion of Deep Fully Convolutional Neural Networks (DFCNN) with Jaccard distance. They employed the 19-DFCNN layer for self-training and the function of the new loss based on the Jaccard scale created by the researchers to re-measure using cross entropy to distinguish the lesion from the skin lesions. The findings of the studies imply that the upgraded classification approach outperforms conventional state algorithms, but that it requires more pre- and post-processing stages for greater accuracy [22]. Using CNN, Xie et al. [23] devised a reliable approach for extracting skin lesion bounds in the existence of distortions in digital images. Due to the use of a basic segmentation strategy, detecting the boundary of a skin lesion zone is slow, but this can be solved by utilizing a semantic segmentation technique [23]. In 2022, the authors trained a feature adaptive transformers network (FAT-Net) and managed to handle blurred boundary issues associated with lesions image. Yet FAT-Net may effectively extract local features and global true label whereas CNN are not capable of learning global true labels sufficiently [24]. Similarly, a neural network-based Multi-scale Residual Encoding and Decoding network (Ms RED) is used to handle blurred boundaries [25]. Thapar et al. [26] employed a segmentation framework using swarm intelligence with Grasshopper Optimization Algorithm (GOA) for feature extraction and successfully obtained 98.42% classification accuracy. Nevertheless, they only trained the model on three skin lesions images [26].

The existence of the nucleus in blood cells is used in determining Leukemia. In 2021, Daud et al. [27] used conventional algorithms such as watershed distance transform and Sobel edge detection algorithm for segmenting nuclei from microscopic images [27]. In another research, authors deployed a Global Local Entropy Histogram Equalization (GLEHE) based segmentation technique to identify Leukemia in blood cells [28]. Dhal et al. [29] provided a method for segmenting blood images for leukemia using the Stochastic Fractal Search (SFS) algorithm, which provides non-false positive segmented results. For image segmentation, the notion of *K*-means-based clustering is studied. The proposed scheme was compared to a previous clustering method, and the findings showed that the system's performance was better in terms of efficiency, computational burden, and quality attributes [29].

Senthil Kumar et al. [30] used five algorithms to extract a plant region from very small lung images, including PSO, inertia-weighted PSO, guaranteed convergence PSO (GCPSO), *K*-means, and *K*-median. The flexible median filter outperformed the central filters, intermediate variables, and standard pre-processing stage, proving that it is best suited for medical CT imaging. In addition, employing the changing histogram balance improves the image brightness. Four algorithms are used to determine the quality of pre-processed images with improved quality. GCPSO has a high accuracy of 95.89 percent when visual results were confirmed with 20 lung sample images using MATLAB [30]. In 2021, van De Worp et al. [31] introduced deep learning-based two-step U-Net architecture for lung cancer segmentation from CT images. Although they performed the task only on 60 CT images [31]. The authors in [32] deployed 2-D Discrete Wavelet Transform (DWT) on the “LOTUS dataset” of lung tumor (CT images) and achieved a dice coefficient of 0.8472.

We give a quick summary of the literature review and the following aspects highlighted as limitations based on the preceding analysis:

- The primary flaw with present clustering-based segmentation methods is that the foreground and background are overlapping.
- Bio-inspired algorithms are commonly utilized in optimization-based techniques, which require longer to complete the segmentation process due to the unknown high number of clusters (Kaushal et al. [33]).
- Because of the image quality, enormous segmentation tasks have suffered from difficulties in segmentation of complex images in cases of computed tomography scans, MRI, microscopic, and dermoscopic image modalities (Kaushal et al. [34]). It is necessary to focus on quality improvisation.
- Researchers encounter a pixel-mixing difficulty due to frequent pixel value changes in the region.

In this study, we are going to make it an intuitive and easy-to-understand framework for medical image segmentation.

## 3. Methodology

This section of the research article includes the procedural and working steps of the proposed model for Medical Image Segmentation using the Traditional Segmentation, SI, and CNN mechanisms. We focused on introducing a modified medical image segmentation approach using CNN as a deep learning and three distinct proposed architectures, which are as given in further sections.

### 3.1. Traditional Segmentation

In this phase, we evaluate the two clustering-based segmentation approaches such as FCM and *K*-means because it has many applications in medical research.

#### 3.1.1. FCM-Based Segmentation

This scenario presents medical image segmentation using the concept of FCM as an unsupervised process. After segmentation, two parts of an image are formed known as the background and foreground part where the foreground is the ROI of any medical data such as MRI, microscopic, dermoscopic, CT-scan, etc. Here, we use some common pre-processing steps in the entire six scenarios of the proposed framework for comparative analysis, first is the color conversion using

(1) $$\begin{array}{c} G_{\text{Image}}=0.3\times +0.59\times +0.11\times , \end{array}$$

where *G*_Image_ is the grey level image that obtained after the color conversion from the color images (RGB ⟶ Red, Green, and Blue plane). After that, grey level mapping is initiated on the clipped region of the image for quality enhancement using the given equation:

(2) $$\begin{array}{c} X_{\text{AVERAGE}}=\frac{X_{\left(\text{region}-\text{xaxis}\right)}\times X_{\left(\text{region}-\text{xaxis}\right)}}{G_{\text{Image}}}. \end{array}$$

Equation (2) defines the average number of pixels in the medical image. Where *X*_(region − xaxis)_ is the total number of medical image pixels in a clipped region (*X*_CLIP_). The clip limit (*X*_CL_) of medical image enhancement is calculated using equation 3 then we apply the image enhancement of the further processing using the written Algorithm 1:

After medical image enhancement in pre-processing phase, we move toward the segmentation using the FCM and the FCM algorithm written as below in Algorithm 2.

The concept of FCM is dependent on the idea of consistently acquiring cluster centers by adjusting their positions using mean values that are given in equation 4 and allows clusters that are more flexible by introducing the possibility of partial memberships. The error function of FCM is written in the following equation:

(3) $$\begin{array}{c} {\text{Error}}_{\text{FCM}}=\sum_{i-1}^{m}\sum_{j=1}^{n}\mu_{ij}^{k}X_{i}^{\left(j\right)}-C_{j}^{2}, \end{array}$$

where fuzzy membership is denoted by *μ*_*ij*_ of *X*_*i*_ (image's pixel) and the cluster identified by its center *C*_*J*_, and here, *k* is a constant that defines the fuzziness of the resulting partitions.

(4) $$\begin{array}{c} \mu_{ij}=\frac{1}{\sum_{m=1}^{C}X_{j}-C_{j}/X_{j}-C_{m}{}^{2/\left(k-1\right)}}. \end{array}$$

The steps involved in the algorithm of FCM-based segmentation are

  FCM 1: recruit Ci as the cluster centers and Iteration *N* = 0
  FCM 2: call FCM membership functions *μ*_*ij*_ according to equation 7
  FCM 3: let *N* = *N* + 1 and assign new *C*_*i*_ as new centers
  FCM 4: until the best convergence is not found, repeat steps 2 to 3.

**Figure 3:**
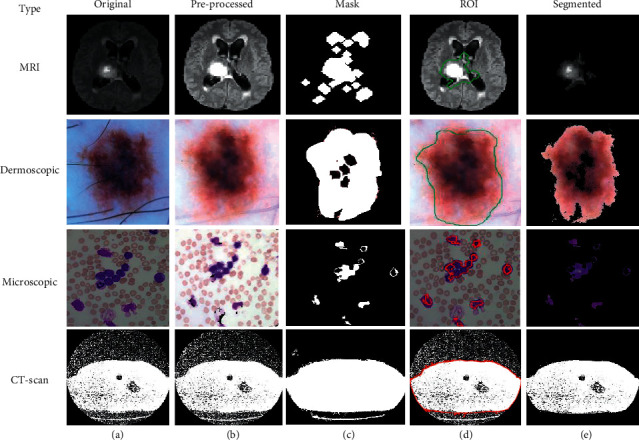
(a) Original. (b) Pre-processed. (c) Mask. (d) ROI. (e) Segmented image using FCM.

Using this algorithm, we segment the ROI from the medical images and after segmentation of medical images; the obtained segmented result with original images shown in Figure 3.

#### 3.1.2. *K*-Means Based Segmentation

**Figure 4:**
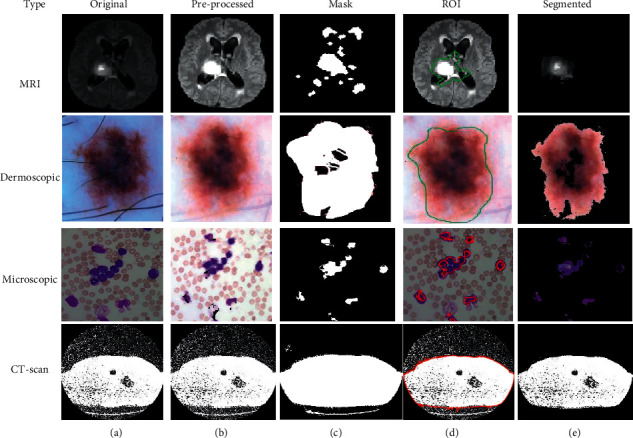
(a) Original. (b) Pre-processed. (c) Mask. (d) ROI. (e) Segmented image using *K*-means.

This is the second scenario and we used *K*-means as a segmentation technique instead of FCM because *K*-means helps to provide better segmentation results as compared to the FCM that is shown in Figure 4. By utilizing the concept of *K*-means as a medical image segmentation technique, appropriate ROI from the medical images could be segmented but also *K*-means faced mix-up issues, and the algorithm of *K*-means is written in Algorithm 3.

Based on the above written *K*-means algorithm in the ASBT system, we obtained better-segmented result as compared to the FCM-based ASBT system, and the results with the original MRI image are shown in Figure 4.

### 3.2. SI-Based Segmentation

In this scenario, we the concept of PSO as a SI approach because it is the most well-known optimization technique that helps to optimize the pixel-mixing problem faced by the FCM. Here, we present two different hybrid mechanisms named FCM and K-means with PSO for medical image segmentation.

#### 3.2.1. FCM with PSO-Based Segmentation

In this scenario, we utilize the concept of PSO along with the FCM as a medical image hybrid segmentation. PSO is a powerful meta-heuristic technique that is favored by birds, mammals, and other insects that live or move in swarms. The best example of PSO inspiration is a flock of birds or a school of tiny fish that helps to reduce the pixel-mixing problem faced by the FCM during medical image segmentation by having very similar neighborhood pixel values. This hybrid segmentation method is based on the participation of individuals of each particle from many fields that are participating in the searching mechanism of a threshold value to minimize the mixed pixels. To solve a medical image segmentation problem, each particle modifies its threshold value based on its own and its neighbors' experiences. Formally, each PSO particle *P*_*I*_ has a position *P*_*I*_ (*t*) at the time *t* instances in the search space, which change at time *t* + 1 by a velocity *V*_*I*_(*t*). In the PSO algorithm, *V*_*I*_(*t*) velocity is influenced by the best position *V*_BEST_(*t*) visited by itself and *P*_ALL_ (*t*) the best position visited by all particles (we termed it “global best”). Each particle's position is determined by a unique fitness function (Fit (Fun)), which is dependent on the segmentation issue and space dimension *D*.

(5) $$\begin{array}{c} P_{I}\left(t\right)=P_{I1},P_{I2},P_{I3},\ldots \ldots P_{ID},\\ V_{I}\left(t\right)=V_{I1},V_{I2},V_{I3},\ldots \ldots V_{ID},\\ V_{\text{BEST}}\left(t\right)=V_{\text{BEST}1},V_{\text{BEST}2},V_{\text{BEST}3},\ldots \ldots V_{\text{BESTD}},\\ P_{\text{ALLI}}\left(t\right)=P_{\text{ALL}1},P_{\text{ALL}2},P_{ALL3},\ldots \ldots P_{ALLD}. \end{array}$$

Kennedy and EberhaVrt, (1995) [9] established the PSO algorithm as an evolutionary image segmentation technique, and the algorithm of FCM with PSO segmentation is written below in Algorithm 4.

**Figure 5:**
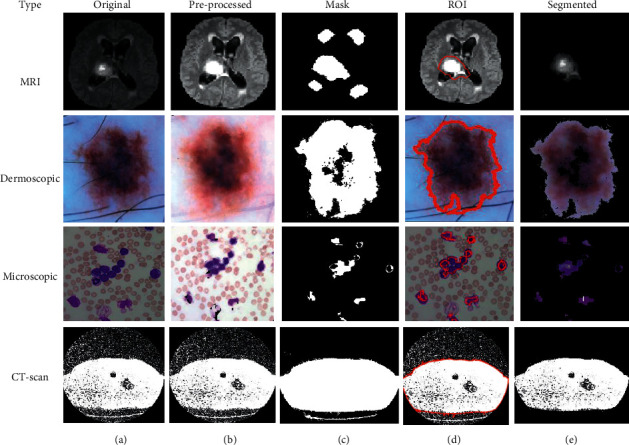
(a) Original. (b) Pre-processed. (c) Mask. (d) ROI. (e) Segmented image using FCM with PSO.

Based on the above-written hybrid segmentation algorithm using FCM with PSO, we obtained better-segmented results as compared to the only FCM as well as K-means also and results with the original medical image shown in Figure 5.

#### 3.2.2. *K*-Means with PSO-Based Segmentation

The concept of PSO along with the *K*-means clustering algorithm used as a medical image hybrid segmentation and the algorithm of *K*-means with PSO segmentation is written below in Algorithm 5.

**Figure 6:**
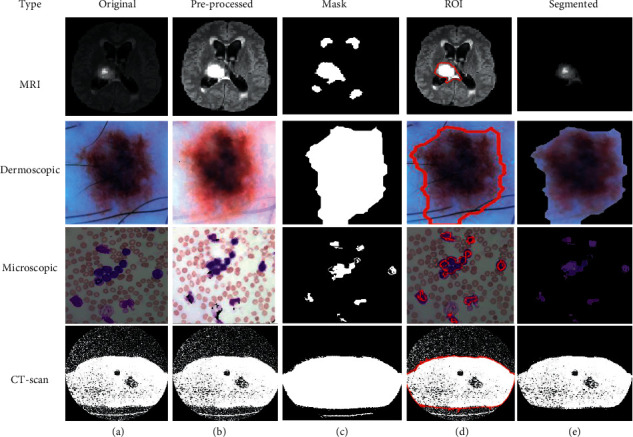
(a) Original. (b) Pre-processed. (c) Mask. (d) ROI. (e) Segmented image using K-means with PSO.

Based on the above-written hybrid segmentation algorithm using K-means with PSO, we obtained better-segmented results as compared to the FCM, K-means, and FCM with PSO also and results with the original medical image shown in Figure 6.

### 3.3. CNN-Based Segmentation

This is the third module of implementation where we used again two different scenarios that are described below.

#### 3.3.1. FCM with CNN-Based Segmentation

**Figure 7:**
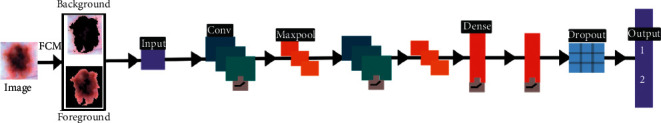
FCM with CNN for segmentation.

In this scenario, we utilize the concept of CNN as a deep learning mechanism along with the FCM as a medical image hybrid segmentation. This hybrid method is currently used in most of the existing medical image segmentation research. First, we train the model using lots of already segmented images in terms of background and foreground images having 3 dimensions (RGB). Usually, segmented medical images, which are fed into the neural network, are reduced in data dimensions, reduce the system processing time as well as complexity and help to reduce the over-fitting problems and hybrid CNN mechanism shown in Figure 7.

Figure 7 illustrated the process of medical image segmentation using the hybridization of FCM with CNN and the algorithm of K-means with PSO segmentation written below in Algorithm 6.

**Figure 8:**
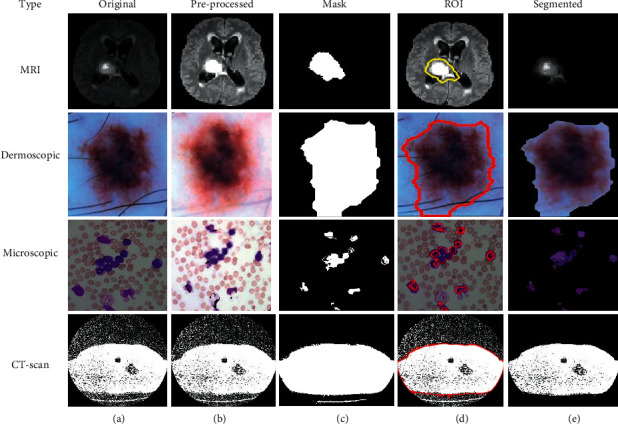
(a) Original. (b) Pre-processed. (c) Mask. (d) ROI. (e) Segmented image using FCM with CNN.

We obtained better-segmented results for the proposed hybrid mechanism of FCM with CNN as compared to the FCM, *K*-means, and improvisation in FCM with PSO, improvisation in *K*-means with PSO and results with the original medical image shown in Figure 8.

#### 3.3.2. *K*-Means with CNN-Based Segmentation

**Figure 9:**
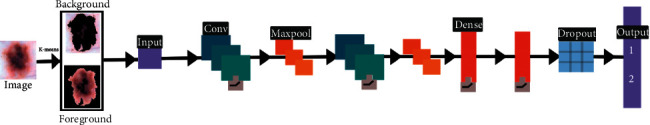
K-means with CNN for segmentation.

The concept of CNN as a deep learning mechanism along with the *K*-means used in this scenario and similar to algorithm 6, a hybrid CNN mechanism with *K*-means is shown in Figure 9.

Figure 9 illustrated the process of medical image segmentation using hybridization of FCM with CNN and the algorithm of *K*-means with PSO segmentation written below in Algorithm 7.

**Figure 10:**
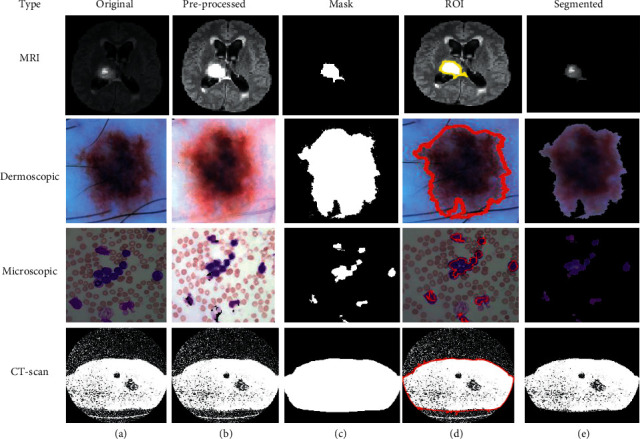
(a) Original. (b) Pre-processed. (c) Mask. (d) ROI. (e) Segmented image using *K*-means with CNN.

In comparison to FCM, *K*-means, FCM with PSO, CNN, and *K*-means with PSO, we achieved better-segmented results for the proposed hybrid mechanism of *K*-means with CNN, and results with the original medical image are displayed in Figure 10.

Finally, performance parameters for different types of datasets are calculated and compared using a comparison framework simulation in terms of Precision, Recall, *F*-measure, Accuracy, Error, MCC, DC, JC, and time.

### 3.4. Collected Dataset

#### 3.4.1. Brain Tumor Segmentation (BraTS) Dataset

**Figure 11:**
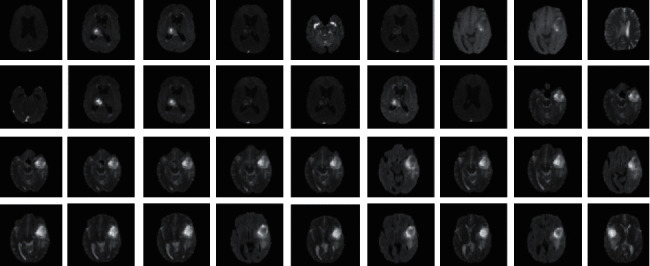
BraTS dataset.

The sample images of the BraTS dataset are shown in Figure 11, it is a standard dataset obtained from “https://www.med.upenn.edu/sbia/brats2018/data.html” having MRI images [35]. For the simulation of the model, 50 DICOM files were converted into JPG format that is representing multi-frame superimposed brain images.

#### 3.4.2. Acute Lymphoblastic Leukaemia Image Database (ALL-IDB) Dataset

**Figure 12:**
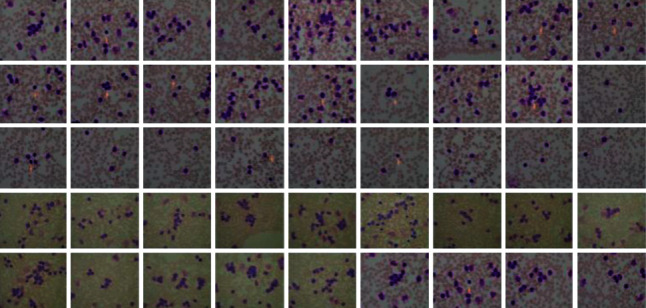
ALL-IDB dataset.

The dataset contains 2008 images that were collected in September 2005 in the Image Processing Department of Computer Science-UniversitàdegliStudi di Milano” [36]. The ALL-IDB dataset of microscopic images is freely available for scientific research purposes from “https://homes.di.unimi.it/scotti/all/” and the sample of ALL-IDB dataset images is shown in Figure 12.

The used dataset contains approximately 39,000 blood counts, and oncologists labeled the lymphocytes. We resize the original microscopic images of blood samples into a size of 256 × 256 and a total of 2000 images were used in this research work.

#### 3.4.3. ISIC-2018 Dataset

**Figure 13:**
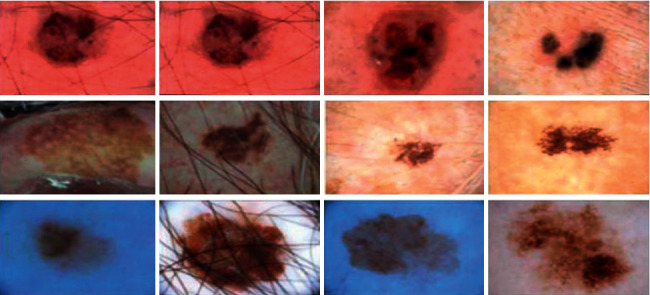
ISIC dataset.

It contains the human lesion analysis toward melanoma detection and the dataset is in the form of dermoscopic images. The dataset is available from https://challenge2018.isic-archive.com/task1/training/ [37]. To capture images, the dermoscopic process is used which is an imaging technique to eliminate the surface reflection of human skin. It provides improved diagnostic accuracy and the sample of the ISIC-2018 dataset is shown in Figure 13.

#### 3.4.4. CT-Scan Dataset

**Figure 14:**
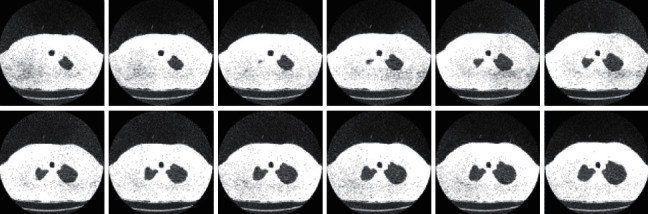
Sample of dataset CT-scan images.

The database currently consists of an image set of 50 lung CT scans for research purposes which is publicly available from “http://www.via.cornell.edu/lungdb.html” [38]. A sample of dataset CT-scan images is shown in Figure 14.

After executing the methodology's outlined steps, the performance has been evaluated in terms of several parameters, as discussed in the result and discussion section.

### 3.5. Evaluation Metric

In this part, we have outlined the assessment measures used to verify the effectiveness of the suggested techniques. First of all, we have observed quantitative metrics such as Accuracy. In most cases, the efficiency of a models is measured in terms of its accuracy. However, in medical image segmentation, the model's accuracy is insufficient to provide a precise understanding of the model. Therefore, there are several additional measures, such as precision, recall, Error, and *F*1 score, to assess segmentation quality. In order to analyze and comprehend the ability of the models, we have made use of each of these measures.

Moreover, we have considered similarity metrics such as Matthews's Correlation Coefficient (MCC), Dice coefficient (DC), and Jaccard Coefficient (JC). Each similar metric has a few special characteristics to evaluate the true performance of the selected segmentation techniques. If all of the probabilistic methods, including true positives, true negatives, false negatives, and false positives, provide a high score, then the MCC algorithm will generate a higher score [39]. Similarly, DC deals with the missing data in image segmentation-related problems [40]. Both quantitative and similar metrics are considered in our study, which provides more robust comparisons and preferences of the specified segmentation techniques [41].

## 4. Results and Discussion

In this research work, we proposed a comparative framework for the medical image segmentation from various types of images such as MRI, Dermoscopic, Microscopic, and CT-scan images using the six different scenarios such as FCM, *K*-means, and improvisation of FCM using PSO, improvisation of *K*-means with PSO, improvisation of FCM with CNN and improvisation of *K*-means with CNN. Simulation results of the offered scenario are shown in Table 1 based on the quantities parameters.

**Figure 15:**
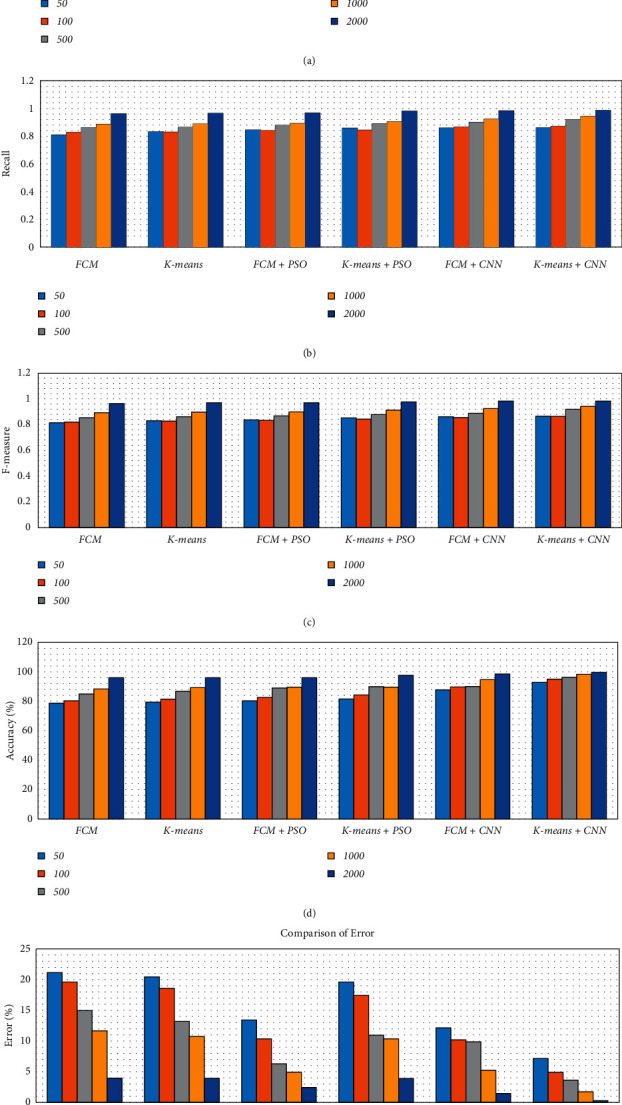
Comparison of simulation results based on quantities parameters. (a) Precision. (b) Recall. (c) *F*-measure. (d) Accuracy. (e) Error.

There are five separate sets of data that included a varied number of images considering 50, 100, 500, 1000, and 2000 images. We have considered an equal number of images from each dataset to produce five specified subsets. Then we applied various segmentation techniques to the subsets of data. The concluded result has been described using different metrics such as precision, recall, accuracy, and F-measure, as shown in Table 1 and Figure 15.

From Table 1 and Figure 15, we observed that the simulation results of proposed frameworks, and hybridization of the K-means with CNN is superior to other modules in terms of the quantities parameters. Improvements in quantities parameters are clearly visible in Figure 15 and average accuracy is 85.72%, 86.06%, 87.54%, 88.64%, 92.21%, and 96.45% for FCM, *K*-means, FCM with PSO, *K*-means with PSO, FCM with CNN, and *K*-means with CNN, respectively. So, we can say that the effect of CNN on *K*-means for medical image segmentation is far better than other combinations. However, we need to validate the model based on similar parameters such as MCC, DC, JC, and computational time.

Therefore, the simulation results based on the similar values have been given in Table 2. In terms of similarities parameters, also *K*-means with CNN is superior to other modules for all the similarities metrics such as MCC, JC, and CD. The required time for *K*-means with CNN is slightly higher than other models. However, the change is extremely minute and may safely be ignored as a result. FCM with CNN is the second most successful segmentation technique based on both quantitative and similarity metrics. Also, it is transparent that the CNN-based optimized segmentation techniques performed better than both swarm intelligence and traditional methods.

Achieving maximum accuracy is the goal of the proposed framework with a fast response to segmentation with pre-processing. The proposed framework offers an extremely self-configurable and standalone mechanism with lots of deep learning interfaces. In addition, our suggested framework generated robust models for segmenting the region of interest in various issues. So, it is a universal framework for medical image segmentation. However, to validate the efficiency of the system, we need to compare it with state-of-the-artwork based on their accuracy in Table 3.

It is clear that the previous study was conducted on a single issue such as skin lesions, brain tumors, lung cancer, or leukemia (as shown in Table 3). However, our study aims to build a universal model for dealing with all the issues. In our case, we have found an average accuracy of 96.45 with *K*-means with CNN.

## 5. Conclusion

In this study, we have introduced a comparative framework for medical image segmentation with traditional, swarm intelligence and convolutional neural networks as a deep learning mechanism. This framework helps to design a real-time universal medical diagnosis system for various types of images such as MRI for brain data analysis, dermoscopic for skin, microscopic for blood leukemia, and CT-scan images for lungs. Here, we present a comparative study using six different scenarios such as FCM, *K*-means, improvisation of FCM using PSO, improvisation of *K*-means with PSO, improvisation of FCM with CNN, and improvisation of *K*-means with CNN. We proved the functionality of *K*-means with CNN is a powerful hybrid mechanism that achieves 96.45% accuracy whereas, other mechanisms achieve 85.72%, 86.61%, 87.54%, 88.64%, and 92.21% FCM, *K*-means, and improvisation of FCM using PSO, improvisation of *K*-means with PSO, improvisation of FCM with CNN and improvisation of *K*-means with CNN respectively. We also found that CNN-based optimized algorithms performed well compared to optimized swarm intelligence and/or traditional methods. We expect that in the future, it will aid in the transition of medical image segmentation from research laboratories to operational or real-time applications.

## Figures and Tables

**Algorithm 1:**
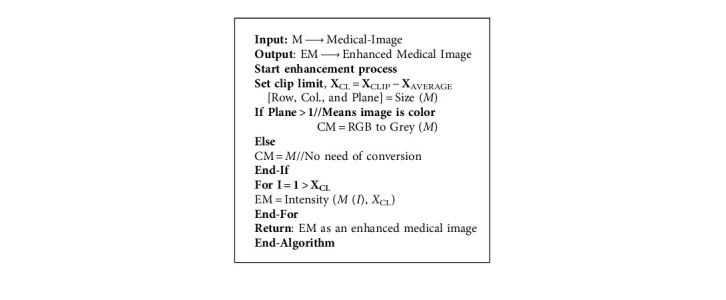
Enhancement of medical images.

**Algorithm 2:**
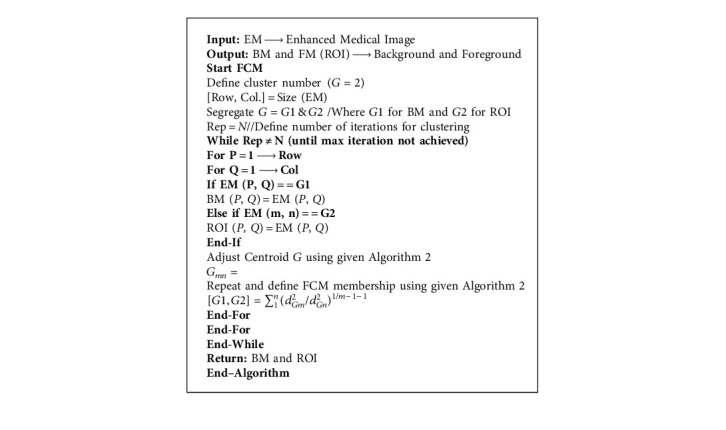
FCM-based Segmentation.

**Algorithm 3:**
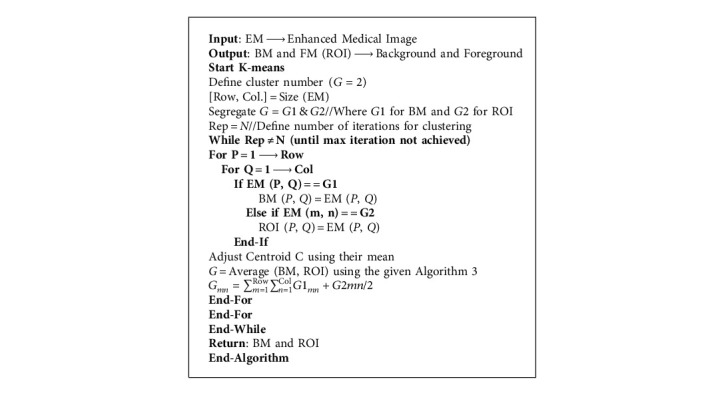
*K*-means based Segmentation.

**Algorithm 4:**
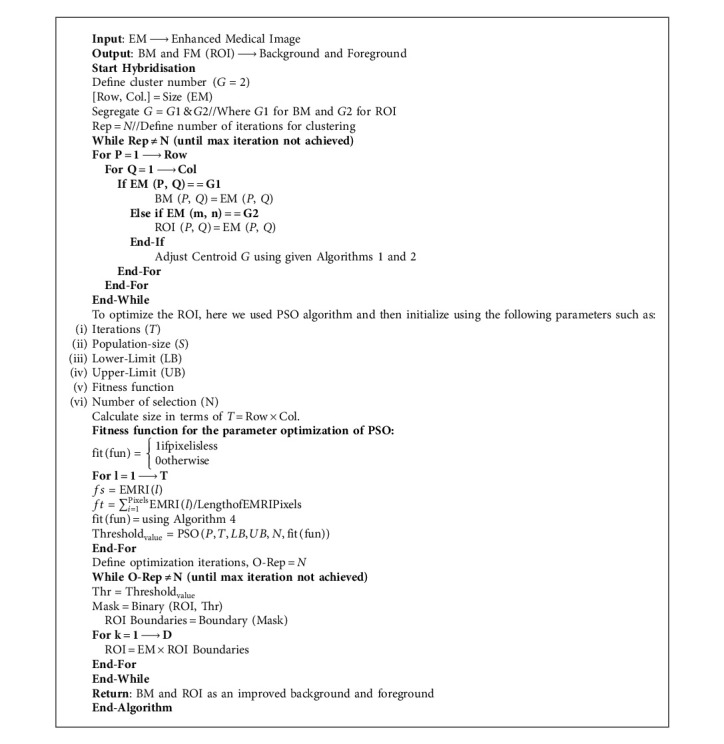
FCM with PSO based Segmentation.

**Algorithm 5:**
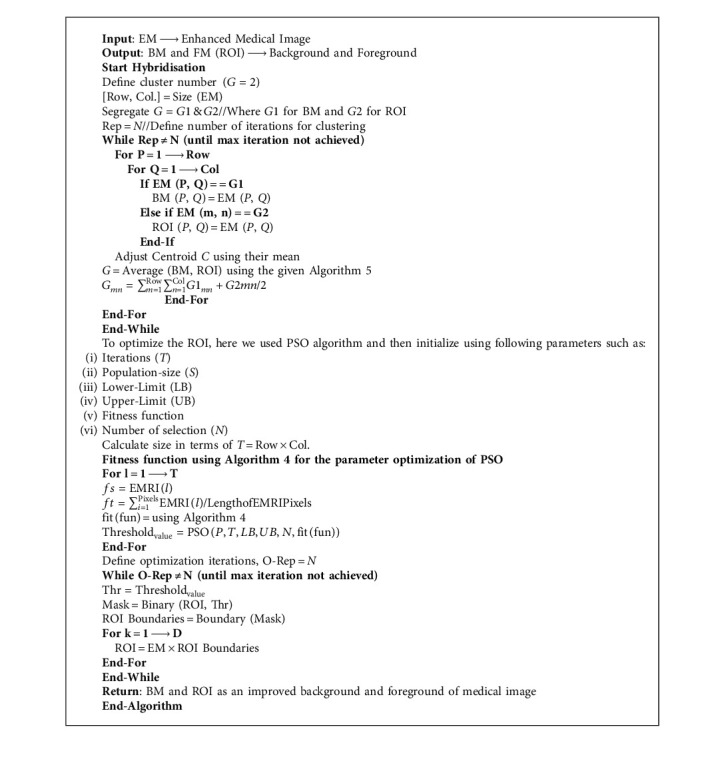
*K*-means with PSO based Segmentation.

**Algorithm 6:**
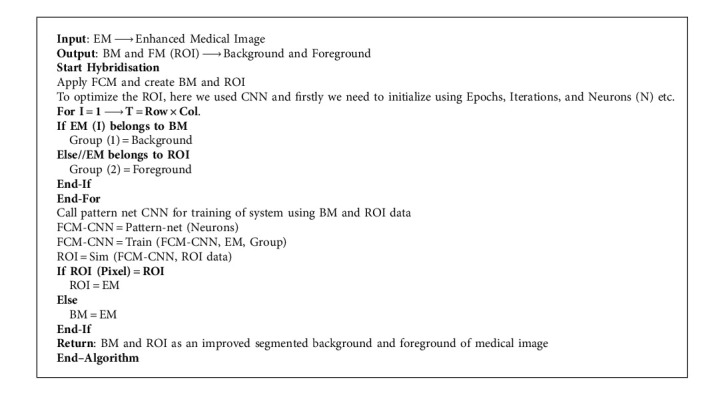
FCM with CNN based Segmentation.

**Algorithm 7:**
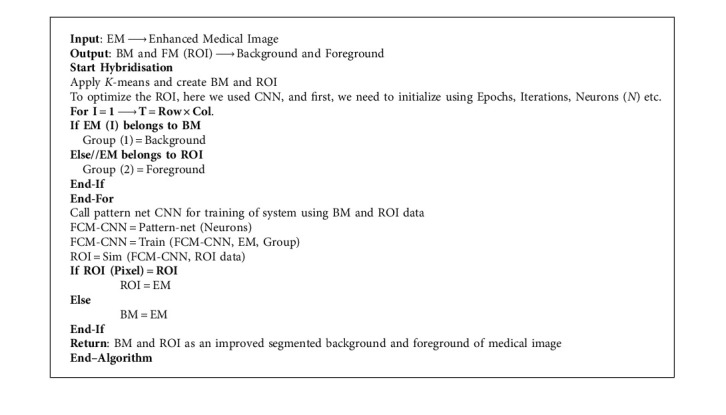
*K*-means with CNN based Segmentation.

**Table 1:** Comparison of simulation results based on quantities parameters.

Parameters		FCM		*K*-means	FCM + PSO	*K*-means + PSO	FCM + CNN	*K*-means + CNN
Precision	No. of images	50	0.8198	0.8291	0.8422	0.8474	0.8627	0.8699
		100	0.8154	0.8261	0.8295	0.8422	0.8448	0.8591
		500	0.8469	0.8583	0.8667	0.8694	0.8765	0.9197
		1000	0.8988	0.9052	0.9138	0.9237	0.9265	0.9431
		2000	0.9647	0.9725	0.9729	0.9737	0.9821	0.9821

Recall	No. of images	50	0.8097	0.8307	0.8441	0.8575	0.8599	0.8611
		100	0.8268	0.8282	0.8392	0.8430	0.8651	0.8701
		500	0.8608	0.8642	0.8792	0.8895	0.8999	0.9190
		1000	0.8864	0.8892	0.8924	0.9037	0.9232	0.9421
		2000	0.9617	0.9661	0.9673	0.9803	0.9831	0.9851

F-measure	No. of images	50	0.8147	0.8298	0.8364	0.8524	0.8612	0.8654
		100	0.8211	0.8271	0.8325	0.8426	0.8548	0.8645
		500	0.8537	0.8612	0.8686	0.8793	0.8880	0.9193
		1000	0.8925	0.8971	0.8987	0.9135	0.9248	0.9426
		2000	0.9631	0.9692	0.9698	0.9769	0.9825	0.9835

Accuracy (%)	No. of images	50	78.8307	79.5256	80.3841	81.5816	87.8346	92.8432
		100	80.3989	81.4237	82.5393	84.3495	89.8007	95.1030
		500	85.0235	86.7798	89.0326	90.0117	90.1153	96.3699
		1000	88.3359	89.2489	89.6378	89.6862	94.7604	98.2569
		2000	96.0569	96.0690	96.1101	97.6035	98.5501	99.6902

Error (%)	No. of images	50	21.1693	20.4744	13.4393	19.616	12.1654	7.1568
		100	19.6011	18.5763	10.3607	17.4607	10.1993	4.8970
		500	14.9765	13.2202	6.2858	10.9674	9.8847	3.6301
		1000	11.6641	10.7511	4.9269	10.3622	5.2396	1.7431
		2000	3.9431	3.931	2.4456	3.8899	1.4499	0.3098

**Table 2:** Comparison of simulation results based on similar parameters.

Parameters		FCM		*K*-means	FCM + PSO	*K*-means + PSO	FCM + CNN	*K*-means + CNN
MCC	No. of images	50	0.7749	0.7917	0.8014	0.8447	0.8893	0.9706
		100	0.7792	0.7999	0.8108	0.8651	0.9157	0.9713
		500	0.8008	0.8216	0.8125	0.8858	0.9394	0.984
		1000	0.8022	0.8305	0.8587	0.8882	0.945	0.9857
		2000	0.8151	0.8656	0.8734	0.9023	0.9485	0.9979

JC	No. of images	50	0.7626	0.8085	0.8047	0.8418	0.8982	0.9668
		100	0.7853	0.8135	0.8092	0.8602	0.8993	0.9717
		500	0.7804	0.8324	0.8416	0.8834	0.9165	0.9793
		1000	0.8184	0.8406	0.8562	0.8912	0.9363	0.9867
		2000	0.8225	0.8562	0.8758	0.8957	0.9489	0.9871

CD	No. of images	50	0.7806	0.8349	0.8433	0.8897	0.8941	0.9608
		100	0.8240	0.8407	0.8528	0.8975	0.8986	0.9610
		500	0.8245	0.8431	0.8638	0.9158	0.9076	0.9695
		1000	0.8546	0.8462	0.8777	0.9221	0.9215	0.9865
		2000	0.8692	0.8879	0.8781	0.9473	0.9272	0.9888

Time	No. of images	50	7.8986	8.17185	8.21999	8.25839	8.45225	8.5419
		100	8.1251	8.12981	8.13375	8.37362	8.50933	8.53367
		500	8.41027	8.47856	8.66652	8.79246	8.83669	9.18777
		1000	8.82637	8.90073	8.92192	9.01242	9.28575	9.4042
		2000	9.60145	9.63701	9.69636	9.74011	9.76711	9.79502

**Table 3:** Comparison with existing works.

Previous works	Accuracy (%)
ANN (artificial neural network) for brain tumor images [17]	94.07
*K*-means clustering with SVM classifier for brain tumor [18]	93.00
CNN for skin lesions [23]	93.80
Swarm-based PSO inertia-weighted PSO for lung cancer [30]	95.81
SVM classifier for skin lesions [42]	94.00
FFBPNN (feed forward back propagation neural networks) for lung cancer [43]	92.60
VGG-SegNet for lung nodules [44]	99.68
*K*-means for brain tumor [45]	94.06
Deep learning with auxiliary task for skin lesions [46]	94.32
U-net and attention U-net for skin lesions [47]	0.913 and 0.913
Semantic segmentation for leukaemia [48]	99.10
Proposed models	Average accuracy (%)
FCM	85.72
*K*-means	86.61
FCM with PSO	87.54
*K*-means with PSO	88.64
FCM with CNN	92.21
*K*-means with CNN	96.45

## Data Availability

The data are available at Brain Tumor Segmentation (BraTS) Dataset: https://www.smir.ch/BRATS/Start2015; Acute Lymphoblastic Leukemia Image Database (ALL-IDB) Dataset: https://homes.di.unimi.it/scotti/all/ISIC-2018; Dataset: https://challenge.isic-archive.com/data/CT-Scan; and Dataset-https://www.ncbi.nlm.nih.gov/pmc/articles/PMC2176079/.
